# Human Neural Stem Cell Systems to Explore Pathogen-Related Neurodevelopmental and Neurodegenerative Disorders

**DOI:** 10.3390/cells9081893

**Published:** 2020-08-12

**Authors:** Matteo Baggiani, Maria Teresa Dell’Anno, Mauro Pistello, Luciano Conti, Marco Onorati

**Affiliations:** 1 Unit of Cell and Developmental Biology, Department of Biology, University of Pisa, 56126 Pisa, Italy; matteo.baggiani@phd.unipi.it; 2 Cellular Engineering Laboratory, Fondazione Pisana per la Scienza ONLUS, 56017 Pisa, Italy; mt.dellanno@fpscience.it; 3 Retrovirus Center and Virology Section, Department of Translational Research, University of Pisa and Virology Division, Pisa University Hospital, 56100 Pisa, Italy; mauro.pistello@unipi.it; 4 Department of Cellular, Computational and Integrative Biology—CIBIO, University of Trento, 38122 Trento, Italy; luciano.conti@unitn.it

**Keywords:** human neural stem cells, TORCH syndrome, microcephaly, neurodevelopment, neurodegeneration, Alzheimer’s disease

## Abstract

Building and functioning of the human brain requires the precise orchestration and execution of myriad molecular and cellular processes, across a multitude of cell types and over an extended period of time. Dysregulation of these processes affects structure and function of the brain and can lead to neurodevelopmental, neurological, or psychiatric disorders. Multiple environmental stimuli affect neural stem cells (NSCs) at several levels, thus impairing the normal human neurodevelopmental program. In this review article, we will delineate the main mechanisms of infection adopted by several neurotropic pathogens, and the selective NSC vulnerability. In particular, TORCH agents, i.e., *Toxoplasma gondii*, others (including Zika virus and Coxsackie virus), Rubella virus, Cytomegalovirus, and Herpes simplex virus, will be considered for their devastating effects on NSC self-renewal with the consequent neural progenitor depletion, the cellular substrate of microcephaly. Moreover, new evidence suggests that some of these agents may also affect the NSC progeny, producing long-term effects in the neuronal lineage. This is evident in the paradigmatic example of the neurodegeneration occurring in Alzheimer’s disease.

## 1. Human Neural Stem Cells: Introduction

The immense complexity of the human brain is reflected in its cellular organization and in the cognitive and behavioral repertoire that defines us as human. The human brain is the product of an evolutionary and developmental history that resulted in its progressive enlargement and specialization. Our central nervous system (CNS) develops through a dynamic and prolonged process in which myriad cell types are generated by neural stem cells (NSCs) and assembled into an intricate synaptic circuitry. Deviations from the normal course of development can lead to a variety of pathologies, including neurological and psychiatric disorders that affect some of the most distinctly human aspects of cognition and behavior [1,2,3,4].

**Figure 1:**
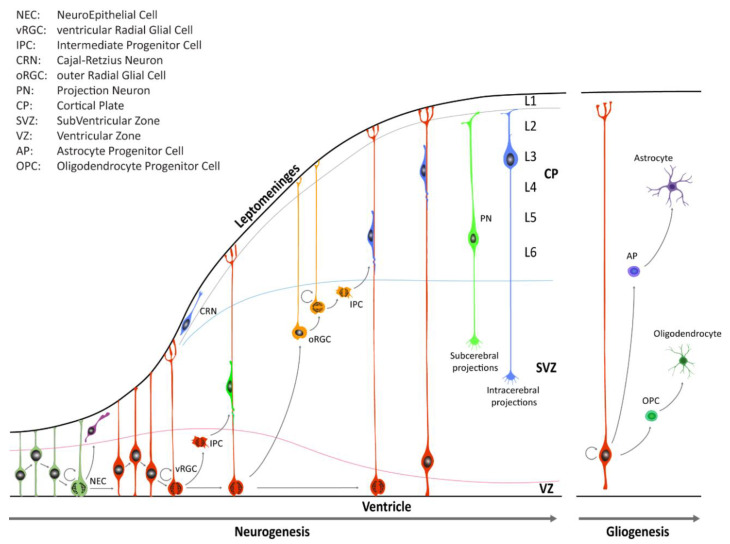
Schematic illustration of neocortical development. Neuroepithelial cells (NECs) undergo symmetric cell division to expand the initial pool and later transition into ventricular radial glia cells (vRGCs). vRGCs begin asymmetric cell division to generate another vRGC and a nascent projection neuron. Neurons then migrates radially from the ventricular zone (VZ) along the RGC basal processes into the cortical plate (CP). Early-born projection neurons (PNs) settle in the deep layers (Layers 5 and 6), and later-born neurons in upper layers. Additionally, some populations of RGC daughter cells convert themselves into intermediate progenitor cells (IPCs) or outer radial glial cells (oRGCs) in the subventricular zone (SVZ). After the neurogenic stages, gliogenesis occurs, generating astrocytes and oligodendrocytes.

During early CNS development, the neural tube is comprised of a pseudostratified layer of neuroepithelial cells lining the central cavity [3,5,6]. These cells constitute the ventricular zone (VZ) of the neural tube and are the founders from which all neurons and glial cells of the adult CNS will be generated (Figure 1).

Until the seventh post-conceptional week (pcw), depending on the region of the CNS, neuroepithelial cells (Box 1) undergo primarily symmetric divisions in order to expand the stem cell pool [3]. Dividing neuroepithelial cells are characterized by a radial movement of cell nuclei from the apical luminal side (apical surface) to the basal side of the neural tube (basal lamina at the pial surface), in concert with the progression of the cell cycle. Mitosis typically occurs when cell nuclei are close to the apical surface of the neuroepithelium, and this continuous relocation is commonly defined as interkinetic nuclear migration (IKNM). Later on, neuroepithelial cells transition into a distinct class of cells known as radial glia cells (RGCs) which reside in the VZ and in the inner and outer subventricular zone (iSVZ and oSVZ, respectively) (Box 1). RGCs contact, at least initially, both the ventricular and pial surface through their apical and basal process, respectively. These cell populations serve as progenitor cells to generate neurons and macroglia (i.e., astrocytes and oligodendrocytes) and to provide a scaffold for migrating nascent neurons [3,5,6]. RGCs divide, but unlike neuroepithelial cells, the divisions of RGCs are mostly asymmetric, giving rise to either a daughter RGC, an intermediate progenitor cell (IPC, also known as a transit amplifying cell, Box 1), or a nascent neuron that subsequently migrates out of the VZ or the SVZ to its final location near the pial surface. In the human neocortex, neuroepithelial cells give rise first to ventricular RGCs (vRGCs), which later transit into outer RGCs (oRGCs) [7,8,9]. oRGCs have morphologically distinct features, retain the basal process, but lose apical contact, and their cell bodies translocate into the oSVZ. The oRGCs can be characterized by a distinct transcriptional signature compared with vRGCs and divide in a unique manner, called mitotic somal translocation (MST), a process where the cell soma moves rapidly up the basal fiber before cytokinesis [8]. A third population of RGCs, called truncated radial glia (tRGCs), develops later in neurogenesis [10]. Cell bodies of tRGCs reside near the apical surface and possess basal processes that do not reach the pial surface and appear truncated. During early neurogenesis, at the beginning of the second trimester, the vRGCs and IPCs give rise to neurons present in the deep layers. On the other hand, oRGCs give rise to later born IPCs, and differentiate predominantly into upper layer neurons (Figure 1). The six cortical laminae are comprised of multiple molecularly defined excitatory neuron subtypes. These cells connect intra-cortically to regulate synaptic activity inside the cortex, as well as subcortically to provide executive regulation of sensory and motor activity [11].

Box 1 **Neural Stem Cells at a glance.**

**Neuroepithelial Cells**: During mammalian embryogenesis, CNS development begins with the induction of the neuroectoderm, which forms the neural plate and then folds to give rise to the neural tube. Within these neural structures there exists a complex and heterogeneous population of neuroepithelial cells, the earliest neural progenitor type to arise. As CNS development proceeds, neuroepithelial cells give rise to temporally and spatially distinct neural stem/progenitor cell populations.

**Radial Glia Cells (RGCs):** Multipotent neural progenitors with glial-like properties. At the onset of neurogenesis, neuroepithelial cells in the ventricular zone transition into RGCs, bipolar cells with long radial processes extending from the apical surface of ventricular zone to the pial surface. RGCs also act as scaffolds along which newborn neurons can travel from their site of origin to their final destination in the adult CNS.

**Intermediate Progenitor Cells (IPCs or Basal Progenitors)**: Neural progenitors generated from neuroepithelial cells and RGCs at the apical surface of the ventricular zone. IPCs migrate to the basal side of the ventricular zone forming the subventricular zone. Each IPC divides symmetrically to generate two or four neurons.

**Adult Neural Stem Cells**: Populations of multipotent neural stem cells mainly present in two specialized niches of the adult mammalian brain, the subventricular or subependymal zone of the lateral ventricle wall and the subgranular zone of the dentate gyrus. They maintain neurogenesis and gliogenesis throughout adult life in rodents and other mammals, but their presence and activity in humans is still debated. They derive from RGCs that in the postnatal brain convert into astrocytic-like NSCs.

Our knowledge of NSCs has been revolutionized as optimized culture protocols have been put in place [12]. Since Reynolds and Weiss (1992) [13] made the landmark discovery that NSCs could be maintained in culture via propagation in free-floating neurospheres, several works have progressively improved systems for NSC long-term and homogeneous culture. In 2005, it has been shown that NSCs can be expanded as monolayer cultures, named NS cells, with full preservation of their neurogenic potential [14]. NS cells were derived from fetal and adult mouse CNS, but also from neural-committed mouse embryonic stem cells (ESCs) and induced pluripotent stem cells (iPSCs) [14,15,16,17,18,19]. Additionally, NS cells were also derived from post-mortem human fetal tissue [20,21]. Although NS cells showed features of neurogenic RGCs, they emerged to be restricted to the generation of GABAergic neurons, independently of the different sources they were derived from [19,20,21].

On the other hand, embryonic neuroepithelial cells possess a great self-renewing potential and wide multilineage differentiation. Thanks to these unique properties, neuroepithelial cells represent an ideal candidate for in vitro studies related to NSC biology, neuronal and glial differentiation, and various neurodevelopmental diseases. Notably, Austin Smith’s group has described a population of NSCs derived from 5–7 pcw human hindbrain [22]. These cells, named hindbrain (hb) neuroepithelial stem (NES) cells, are neurogenic and preserve their original regional identity, exhibiting for the first time a stable wide degree of plasticity.

More recently, we described the derivation and characterization of neocortical (NCX) NES cells [23]. NCX-NES cell lines were derived from primary neuroepithelium of human post-mortem specimens ranging from 5 to 8 pcw. After derivation, NCX-NES cells form neural rosettes, reminiscent of the radial arrangement in the native neural tube. NES cells exhibit stem/progenitor cell characteristics as they express the neuroepithelial marker SOX1 and the pan-neural stem cell markers Nestin, SOX2, and Vimentin. NES cells retain regional identity after long-term expansion, as demonstrated by the expression of FOXG1 and OTX2, key transcription factors demarcating proliferative zones of the early human forebrain. NES cells show great neurogenic potentials, giving rise to mature neurons with extended complex neurites. Furthermore, they generate GFAP-positive astroglial cells, thus demonstrating their multipotential stem cell capacity. We also reported that NCX-NES cells could be expanded for more than 1 year and 38 passages with no evidence of chromosomal instability [23]. Single-cell RNA-sequencing (RNA-seq) on expanded NCX-NES cells and cells from donor-matched brains demonstrated that the majority of cells from the donor-matched brain tissue samples express canonical marker genes of neuroepithelial cells and RGCs of the dorsal forebrain [23]. Remarkably, NCX-NES cells exhibit a close transcriptional signature of early NSCs as their donor-matched genetically identical NCX cells. Together, these data establish NES cell lines as a consistent model of early human brain development. A similar NES population derived from developing human spinal cord has been described, able to maintain regional identity and neuronal commitment of the caudal CNS [24]. Of note, spinal-cord NES cells were successfully tested in cell grafting approaches after spinal cord injury in mice [24].

Human pluripotent stem cells (hPSCs), which include both ESCs and iPSCs, represent an extraordinary ex vivo source of neural progenitors. The development of neural induction protocols provides the possibility to generate in vitro-derived expandable NSC systems as a platform for studying basic human neurodevelopment, disease mechanisms, and potential therapeutics [12]. Seminal studies have identified rosette-type NSCs that resemble neural tube-stage progenitors, capable to respond to patterning instructions, but not long-term expandable [25]. On the other hand, a long-term population of NES cells (named lt-hESNSCs or lt-NES) was described by Koch et al. (2009) [26]. The study used the embryoid body (EB)-based differentiation protocol and required manual isolation of the rosettes from plated EBs. However, in vitro culture conditions bias lt-NES regional identity from rostral (first five passages) to more caudal midbrain-hindbrain identity (later passages) [26]. In this direction, Li et al. (2011) [27] described a small molecule-based neural induction method to derive primitive neural progenitors from hESCs. However, also in these conditions, the NSC population is biased towards a midbrain/hindbrain neural fate [27]. A more recent elegant work improved the isolation and propagation paradigm of neuroepithelial and RG-like cells [28], but the true identity and physiological relevance of hPSC-derived NSCs are open to interrogations because cells could acquire transcriptional and epigenetic programs that diverge from the cell state in vivo.

In this review, we will highlight NSCs as the main target of insults that affect human neurodevelopment. In particular, we will propose two different levels at which NSCs are more susceptible: (i) cell cycle machinery; (ii) differentiation program and final neuronal and glial output.

Special emphasis will be given to human NSC model systems employed to unravel mechanisms perturbating human neurodevelopment and/or producing neurodegeneration.

## 2. Neural Stem Cells and Neurodevelopmental Disorders

### 2.1. Genetic and Environmental Insults to NSCs

The brain is the most complex and enigmatic organ of our body, a universe of neuronal connections that defines our human nature. As a result of its extended development, our CNS is susceptible to a host of genetic and environmental insults that might disrupt normal developmental trajectories.

A plethora of insults that targets specifically NSCs at different levels can result in severe neurodevelopmental defects, producing structural, behavioral, and cognitive deviations. Defects of cerebral cortical development have been of interest to clinicians and neuroscientists for many decades. In 1996, the term malformation of cortical development (MCD) was introduced to designate collectively a group of disorders in children with developmental delay and epilepsy. Microcephalies are MCD caused by abnormal neuronal and glial proliferation or apoptosis [29] and are clinically defined as a reduction of occipital-frontal head circumference (OFC) more than two standard deviations below the ethnically-, age-, and sex-matched controls [30], and imply a reduced brain size (micrencephaly). Currently, microcephaly is incurable. It is thought to be the result of a depletion of the founder populations of NSCs in the developing brain, either through cell death or premature differentiation [29,31]. The etiology of microcephaly can be broadly divided into an environmental and genetic landscape:

i. Distinct genetic lesions are involved in microcephaly, usually with a recessive inheritance pattern. Some of the genes that cause microcephaly are responsible for the control of crucial aspects of neural development and may also be evolutionary involved in the expansion of the cerebral cortex that characterizes primates. To date, 18 loci (*MCPH1-MCPH18*) have been implicated in the autosomal recessive form of primary microcephaly (MCPH). Among them, *ASPM*, *WDR62*, and Microcephalin 1 (*MCPH1*) have been linked with cerebral cortical development, proliferation, and migration of neuronal precursors (for review see Naveed 2018 [32]).

ii. Common environmental causes include congenital infections that affect the brain. The rapid spread of Zika virus (ZIKV) and its association with abnormal brain development constitute a recent global health emergency [33]. The disorders caused by ZIKV infection, together with microcephaly, have been called ‘Congenital Zika Syndrome’, and are reminiscent of those that result from congenital infections caused by TORCH syndrome pathogens (see below) [34], which are thought to result in up to half of all perinatal deaths and microcephaly worldwide, with an especially large burden in developing countries [35].

Both, genetic or TORCH-related lesions induce NSCs to enter into a block of cell division, leading either to their premature differentiation or directly to death. This event inevitably compromises the pool of progenitors/NSCs and their neural progeny.

### 2.2. TORCH and Microcephaly

Among the environmental insults that can severely affect brain development, special attention has been paid on TORCH agents. These include teratogenic and neurotropic pathogens that can infect the fetus either by vertical transmission, i.e., from the mother, or immediately after birth. TORCH is an acronym standing for *Toxoplasma gondii*, others (including ZIKV, Coxsackie virus, *Listeria monocytogenes*, *Treponema pallidum*, varicella zoster virus, human immunodeficiency virus (HIV), and parvovirus B19), Rubella virus, Cytomegalovirus, and Herpes simplex virus. TORCH pathogens are thought to cause 50% of prenatal deaths and some brain malformations such as congenital microcephaly (Table 1) [29,30].

### 2.3. TORCH Pathogens Effects on Self-Renewing NSCs

#### 2.3.1. Zika Virus

Zika virus (ZIKV) is a member of the *Flavivirus* genus of the *Flaviviridae* family. It is an enveloped virus and contains a single strand-positive sense RNA genome. This virus is transmitted to the human host preferentially through infected *Aedes* mosquito bite. ZIKV can be also vertically transmitted from pregnant women to the fetus, thus triggering, in most symptomatic cases, the “ZIKV syndrome” that converges in severe neurodevelopmental disorders [86,87].

After the 2015 outbreak of ZIKV in Latin America, several studies have used different in vitro human neural models to investigate ZIKV tropism for neural stem/progenitor cells. Since the huge amount of literature present, we will mention only some contributions (for more exhaustive reviews please see [41,88,89]). Among the firsts, Tang and colleagues used iPSC-derived NSCs to show that ZIKV leads to cell cycle dysregulation and cell death [39]. Other groups used iPSC-derived forebrain organoids cultured in miniaturized spinning bioreactors [40]. Similarly to what was reported in 2D-cultures, ZIKV infection of 3D organoids revealed a specific preference for NSCs with consequent cell death, proliferation reduction, and decrease of neuronal cell-layer volume, an event reminiscent of what happens in microcephaly-affected brains [40].

**Figure 2:**
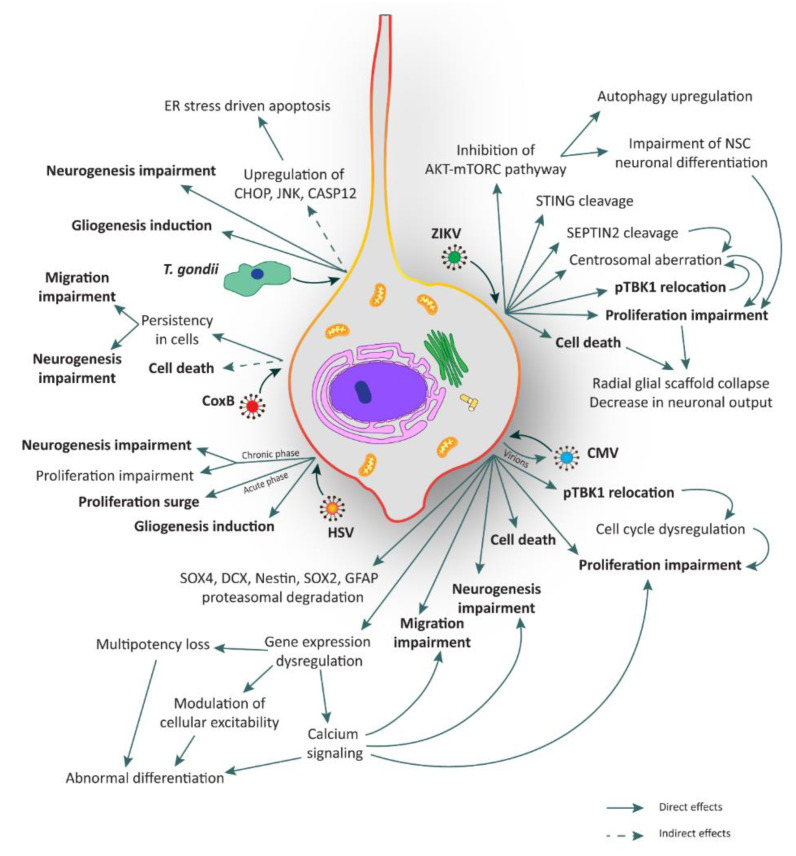
Illustration of the major consequences elicited by TORCH infection in human neural stem cells (hNSCs). hNSCs can be infected by several TORCH pathogens such as Zika virus (ZIKV), Cytomegalovirus (CMV), Coxsackie B virus (CoxB), *Toxoplasma gondii* (*T. gondii*), and Herpes simplex virus (HSV). All these agents can lead to several and critical consequences such as cell death, proliferation impairment, unbalance of neurogenesis and gliogenesis, and migration impairment. Processes in bold are shared among TORCH pathogens. ER: endoplasmic reticulum.

The mechanisms underlying ZIKV infection in NSCs are currently under investigation. Nonetheless, various mechanisms have already been elucidated. We have recently reported that ZIKV infection occurs through the involvement of the TBK1 protein and its active form phospho-TBK1 (pTBK1) [23]. TBK1, which stands for Tank Binding Kinase 1, is a homodimeric serine-threonine kinase involved in many important pathways such as natural anti-viral immune response (activating type-I interferon), inflammation, autophagy, xenophagy of bacteria, as well as cell proliferation, cell growth, and insulin signaling [90,91]. TBK1 is normally present in the cytosol, but during mitosis it is phosphorylated and localizes to the centrosomes [92]. In general, when a virus infects a cell, its RNA o DNA genome is recognized by specific proteins like Toll-like receptor 3 (TLR3), retinoic-acid inducible gene I (RIG-I), or melanoma differentiation-associated protein 5 (MDA5). These proteins directly or indirectly activate TBK1 which, in turn, interacts with some adaptor or scaffold proteins like mitochondrial antiviral-signaling protein (MAVS) or the stimulator of interferon genes (STING), localized in specific compartments like mitochondria or endoplasmic reticulum, respectively [91,92,93]. Using human NCX NES cells, we found that upon ZIKV infection, pTBK1 is relocated from centrosomes to mitochondria, impairing cell cycle progression, correct centrosomal number, and inducing cell death (Figure 2). Additionally, in the same study we reported that ZIKV infects mature neurons with less efficiency than proliferating NES cells [23]. By applying ex vivo human fetal organotypic brain slices, we and others demonstrated that RGCs are the primary cell type infected by ZIKV [23,94]. Intriguingly, ZIKV infection causes radial scaffold disorganization and architectural impairment, thus further contributing to worsening the neurological defects [23].

Recently, Ding and colleagues have highlighted the role of ZIKV proteases in the cleavage of human STING, a scaffold protein involved in the positive regulation of interferon response interacting directly with pTBK1 [37]. Moreover, Li and colleagues revealed that ZIKV mediates the cleavage of SEPTIN2, a protein involved in the cytokinesis at the level of midbody in dividing NSCs [38], thus contributing to mitosis impairment.

Another interesting mechanism of ZIKV neuropathogenesis involves mTORC and autophagy [36]. Indeed, the control of autophagy induction is essential for ZIKV replication. For this reason, the cooperation of NS4A and NS4B (two non-structural (NS) proteins of the virus) has been shown to strongly suppress host AKT-mTORC signaling, a pathway involved in inhibition of autophagy, leading to the impairment of human NSC neuronal differentiation and the upregulation of autophagy for viral replication (Figure 2) [36].

All these strategies are believed to converge on aberrant embryonic NSC proliferation leading to premature differentiation, disruption of the glial scaffold and microcephaly (Figure 2).

Noteworthy, ZIKV has been shown to infect adult individuals although this infection is mostly asymptomatic, sometimes comparable to flu symptoms [39] or dengue fever (red eyes, joint pain, encephalitis, and rash) [95,96]. Nevertheless, rare serious complications due to adult infection of ZIKV have been reported. These include meningoencephalitis and Guillain–Barré syndrome (GBS) which will be discussed below.

#### 2.3.2. *Toxoplasma*

*Toxoplasma gondii* is an obligate intracellular parasitic protozoan belonging to *Sarcocystidae* family. Toxoplasmosis is one of the most common parasitic infections in humans (30–70% of the human population) and is mostly asymptomatic. However, primary infection in a pregnant woman can cause severe and disabling effects on the developing fetus [97]. Hydrocephalus or microcephaly, hepatosplenomegaly, jaundice, convulsions, chorioretinitis (often bilateral), cerebral calcifications, and abnormal cerebrospinal fluid are the classic consequences of severe congenital toxoplasmosis [97]. The life cycle of *T. gondii* is divided into sexual (feline infection) and asexual (non-feline infection) stages. The sexual stage occurs when *T. gondii* infects the epithelial cells of the cat’s small intestine. Inside these intestinal cells, the parasites undergo sexual development and reproduction, producing zygote-containing cysts known as oocysts. Felines are the only definitive host because they lack expression of an enzyme (delta-6-desaturase) responsible for linoleic acid conversion and its absence results in systemic linoleic acid accumulation. Recent findings showed that this excess of linoleic acid is responsible for *T. gondii* sexual reproduction [98]. On the other hand, the asexual stage consists of two distinct growth phases: rapidly replicating tachyzoites and slower growing bradyzoites. After infection, the tachyzoites replicate inside the host cell until they lyse the cell and exit to infect surrounding cells [99,100]. When the host’s immune response begins to fight and eliminate the parasite, tachyzoites differentiate into bradyzoites and form cysts, which can persist for life, typically in the host’s brain, skeletal or cardiac tissue. In a host neuronal cell, cyst generation begins soon after invasion. This phenomenon of spontaneous cyst formation is not observed in non-neuronal cells such as fibroblasts or other neural cells such as astrocytes [101]. Moreover, it has been proposed that the intracellular parasites migrate to the brain as early as 7 days post-infection and cross the blood–brain barrier (BBB) by a Trojan horse mechanism.

For what concerns the relationship between *T. gondii* pathogenesis and the consequences of the infection on human NSCs, very little is known to date. Few studies have explored this issue on mouse NSC systems. One of these works indicates that *T. gondii* promotes mouse NSC apoptosis in a load dependent manner within 24 h post-infection [45]. Upregulation of proteins such as CHOP, JNK, and CASP12, involved in apoptotic events triggered by endoplasmic reticulum stress, has also been described. Whether these changes are mediated by the activation of a death receptor or by a mitochondrial apoptotic signal pathway remains to be elucidated [45,102]. In another study, mouse NSCs were used to test resveratrol to rescue the neurogliogenesis after infection, showing an increase of apoptosis and alterations in migration probably due to a commitment bias towards gliogenesis (Figure 2) [44].

#### 2.3.3. Rubella Virus

Rubella virus (RV) is an enveloped, positive-sense, single-strand RNA virus (genus *Rubivirus*, family *Togaviridae*). RV infection in utero is the leading cause of congenital rubella syndrome (CRS) [103]. An estimated 100,000 cases of CRS occur worldwide each year with a mortality rate for infants up to 33%, and a percentage of miscarriage of about 20% of cases.

The cellular mechanisms responsible for the multiple congenital defects in CRS are currently poorly understood and only few studies have investigated these processes. Although NSCs are the main target, most of the studies have focused on histopathological analyses made on autopsies of aborted or dead fetuses [55]. Epithelial cells and mononuclear progenitor cells in multiple organs and neuronal cells in cerebral cortex were reported to be the predominant affected cell types. Moreover, in a CRS case the presence of underdeveloped granular layer of cerebellum has been described [104,105]. Studies on human glial cells suggested that the main cell type permissive to RV infection in developing brain tissue is represented by astrocytes [106]. Noteworthy, pathological studies on the brain of infants with CRS revealed extensive degenerative changes in neural and vascular tissue [56]. In particular, foci of necrosis localized in the deep white matter and nuclei (the basal ganglia and thalami), but no developmental malformations or significant inflammation of the nervous system were found [56,57].

Thanks to mass vaccination, CRS has been eliminated in the Americas. However, RV still remains a problem in many countries without effective vaccine programs [41]. Indeed, RV infection of progenitor cells could lead to a variety of malformations of the CNS, like developmental delay and microcephaly that are frequently reported in infants with CRS. At present, there are no studies on RV using NSCs in vitro models.

#### 2.3.4. Herpetic Viruses

##### Cytomegalovirus

Human cytomegalovirus (CMV) is a double-strand DNA virus, member of *Herpesviridae* family and *Betaherpesvirinae* subfamily. Approximately 70–100% of healthy adult population is CMV seropositive and the current global estimate for congenital CMV infection is 0.3–0.7% of all live births [107,108] with a risk of congenital CMV transmission highest in pregnant women with primary CMV infection. Up to 50% of CMV infections has non-specific clinical manifestations, while approximately 11% of infants are symptomatic. The infection can cause severe long-term impairments, like hepatosplenomegaly, petechiae, microcephaly, intrauterine growth restriction, progressive sensorineural hearing loss, developmental delay, and death [109]. CMV can also be extremely hazardous for HIV/AIDS patients, immunocompromised individuals, and transplant recipients, for whom the consequences of CMV infection can cause severe diseases and be life-threatening [110].

CMV replication cycle includes the expression of three gene groups temporally coordinated. After the fusion with host cytoplasmic membrane, the capsid inoculates viral DNA in the nucleus through nuclear pores. The first group of genes expressed are immediate-early (IE) genes that arrest host natural immune defense and deregulate the host cell cycle, first, by inducing its progression and then by blocking it before S phase [111]. At this stage, the virus can persist in a latency phase or undergo productive infection. In the latter case, IE genes are required to activate the expression of viral Early (E) and Late (L) genes, involved in viral DNA replication and capsid assembly, respectively [111,112]. Unfortunately, vaccines to CMV do not exist and available drugs mostly target viral DNA polymerase and present several limitations including long-term toxicity, unfavorable pharmacokinetic properties, and emergence of viral resistance. For these reasons, several studies are looking for new therapeutic targets [113,114].

NSCs can be infected by CMV and the main site of infection in the mouse model is the SVZ. Upon infection, NSCs express the full range of viral antigens, release virions [59,60,61,115,116], and display quick and abnormal differentiation. This phenotypic change requires active viral transcription and rapid downregulation of genes that maintain multipotency. Infection of NSCs with CMV results in impaired cell proliferation [59,60,61] and increased cell death [59,61,62,63] which can both occur also in the adult NSC niches, thus confirming that susceptibility to CMV is not lost with aging [116].

Microcephaly and polymicrogyria are the most prominent features of brain disorders associated with congenital CMV infection in humans [61,117,118,119]. One hypothesis is that these brain disorders originate from the disturbance of cellular processes within the ventricular regions. Recent works by Sison and colleagues [120] and Sun and colleagues [58] used human iPSC-derived brain organoids to demonstrate that the virus impairs cell growth in 3D aggregates, leading to both reduced proliferation and increased apoptosis. Moreover, a reduction of cortical layer thickness, probably due to neurogenesis and migration impairment, was also described [58]. Another important aspect is the alteration of calcium signaling, which plays a pivotal role in NSC processes, including proliferation, communication, differentiation, and migration. Low calcium levels are initially required to keep NSCs in a migratory state (Figure 2) [121,122,123,124,125,126,127,128]. Sun and colleagues [58] reported the downregulation of three genes involved in calcium signaling in CMV-infected organoids, namely *ENO2*, involved in calcium modulation in neurons [129]; *BNIP3*, important in endoplasmic reticulum-mitochondria calcium homeostasis [130]; *PDK1*, responsible for calcium entry into cells [131]. Finally, Sun and colleagues also demonstrated the importance of EGFR and PDGFRα receptors for the CMV entry into the cells and the variability of detrimental effects exerted by CMV in human brain organoids depending on the viral strain [58].

CMV infection in vitro can perturb NSC multipotent state through the downregulation of several marker proteins, including SOX4, DCX, Nestin, SOX2, and GFAP, caused by increased proteasomal degradation [60,64]. Moreover, there are several studies regarding the capability of CMV to induce premature differentiation, both in the neuronal and astrocytic lineages. Luo and colleagues hypothesize that CMV infection of NSCs prompts neuronal fate commitment without accomplishing full differentiation and causing development of an abnormal state (Figure 2) [64]. The dynamics of CMV infection in NSCs and its consequences appear to depend on whether the induction is towards the neuronal or the astrocytic lineage. In particular, in the prospective glial cells, viral expression is initially retained, but after few days, it progressively declines. On the other hand, the differentiation into neurons leads to an active repression until total virus disappearance [61,115]. Additionally, in human NSCs, CMV causes dysregulation of genes involved in the modulation of cellular excitability, including tyrosine hydroxylase (*TH*), glutamate decarboxylase (*GAD1*), and the potassium voltage-gated *KCNQ2* [65].

On the other hand, Belzile and colleagues (2014) [132] proposed an opposite study in which CMV infection on human NSCs leads to selective expression of virus immediate-early genes (IE) without a progressive infection, speculating that NSCs could represent a reservoir for the virus. In order to explain the discrepancy, the authors hypothesize that human NSCs used in their work were less gliogenic, as attested by the poor GFAP expression. GFAP-positive astroglial progenitors are more permissive to infection compared to neurogenic precursors [132].

In the above mentioned study, Onorati and colleagues also used Dengue virus and CMV to understand if the pTBK1 re-location was a specific aspect of ZIKV infection. It was found that both viruses can infect NES cells, but only CMV leads to mitochondrial re-location of pTBK1, suggesting that this mechanism may represent a common axis involved in microcephaly (Figure 2) [23].

##### Herpes Simplex Virus

Herpes simplex virus (HSV) is a neurotropic double-strand DNA virus, member of *Herpesviridae* family and *Alphaherpesvirinae* subfamily, with a replication cycle similar to the previously described CMV [133]. Worldwide, the global prevalence of HSV infection is about 90% with an average of approximately 65% in the USA [134] and 52–67% in northern Europe [135]. Neonatal HSV infections occur at a relatively high frequency (approximately one in 3000 births in the USA), and up to two-thirds of infected subjects develop encephalitis. The decrease in viral neurovirulence with age correlates with a decline in brain thymidine kinase activity, an enzyme involved in cell proliferation [136]. There are two species belonging to *Simplexvirus* genus, Herpes Simplex Virus 1 (HSV-1) and Herpes Simplex Virus 2 (HSV-2). Both viruses are extremely contagious, neurotropic, and there is no cross-protection, i.e., an individual can be infected with both species. HSV-1 usually causes herpes labialis or cold sores and HSV-2 genital herpes, but it is not rare the opposite, as well as other disease manifestations [137,138]. Both HSVs are capable of establishing lifelong persistent infection in the host, a condition termed latency. During latent infection, no infectious virus is produced from infected cells, symptoms are not detectable and transmission does not occur [136].

HSV entry into a host cell requires envelope viral glycoproteins and occurs via membrane fusion or endocytosis [139]. Glycoprotein D (gD) seems to be the most important factor thanks to its capacity to bind heparan sulfate and herpes virus entry mediator (HVEM) receptor A (HveA), nectin-2 (HveB), or nectin-1 (HveC) receptors expressed by the host cell plasma membrane. It seems that HSV-2 infects neurons through the HveC receptor forming a latent and immunologically privileged reservoir of infection in the brain [140,141]. Primary HSV infection occurs at the level of oral or genital mucosa, then the virus infects the neuronal dendrites of sensory ganglia through retrograde microtubule-associated transport to the neuronal cell body. At this point, the virus encounters a ‘choice’ of gene expression programs that will lead to either lytic replication or latent infection. The latent virus genome is stably retained within sensory neurons and is characterized by repression of all viral lytic genes. In response to a variety of diverse stimuli, the virus can periodically reactivate to resume viral replication and produce infectious viral particles, which are transported anterogradely back to the periphery to facilitate epithelial cell infection and consequent transmission that triggers reactivation [136].

The mechanism of HSV penetration into the brain and its preference to infect specific regions is currently not well defined. Intracerebrally inoculated HSV mainly disseminates either around the site of injection or spread via retrograde transport to distant brain areas. In some cases, the virus first infects the meninges and ependymal cells and subsequently spreads to areas adjacent to the ventricles [142]. In order to investigate HSV neurotropism, mice organotypic brain slices have been exploited [142]. Neonatal brain tissues showed restricted infection confined to leptomeningeal, periventricular, and cortical brain regions. The hippocampus is also infected, thus supporting that the infection is localized to progenitors and ependymal cells. Neonatal brain is much more permissive for HSV infection than adult brain [142]. Most of the infected cells are SVZ neural precursors. Similar results were obtained by Sun and co-workers who highlighted the higher permissiveness of murine NSCs to HSV-mediated infection [143]. Rotschafer and colleagues pointed out that infection also takes place in adult NSCs, where HSV-1 modulates the proliferation rate of the cells in opposite ways depending on the phase of the infection process [66]. Apparently, in the initial phase, HSV fosters NSC proliferation, whereas mitosis is impaired in the subsequent chronic phase. The authors speculate that the virus propels proliferation as an infective strategy. However, during latency, the persistent inflammation decreases NSC proliferation and impairs neurogenesis (Figure 2) [66]. More studies need to be performed to better understand the mechanism of HSV infection in NSCs.

#### 2.3.5. Coxsackie Virus

Coxsackie (Cox) are a specific group of Enteroviruses (EV) of the *Picornaviridae* family, with small, non-enveloped, single-strand positive RNA [144]. EVs can frequently target human CNS where they induce a variety of neurological diseases like aseptic meningitis and encephalitis, predominantly in very young people. EVs are highly cytolytic and can rapidly shut down the host transcriptional and translational machinery inhibiting the host cell metabolism. Moreover, EV infection can persist and cause a variety of chronic diseases, including myalgic encephalomyelitis/chronic fatigue syndrome and dilated cardiomyopathy [145,146,147].

The mechanism of EV persistence is unclear. Several in vitro studies point to a coevolution of both host cells and viruses. Induced cellular responses (mutational alterations of the receptors) can inhibit virus replication and spread, but viral variants with enhanced infectivity counteract the host responses, leading to the establishment and maintenance of persistence [82,144]. Coxsackie B viruses (CoxB) are distinguished in six serotypes and their RNA can persist for dozens of months in skeletal muscle or the CNS through the formation of a stable dsRNA complex [144].

In neonatal mouse models, proliferating SVZ progenitors have been shown to represent the primary target cells during early CoxB infection. Virus production and protein expression levels are sustained in undifferentiated NSCs, while differentiated cells appear to be refractory to infection and virus protein expression, even if CoxB can persist in quiescent subgroups of NSCs [82]. Like all EVs, CoxB is cytolytic but can accumulate mutations during replication that slow viral replication and attenuate its virulence [85,148,149]. CoxB targets host cells through two main receptors: decay-accelerating factor (DAF), and murine coxsackievirus and adenovirus receptor (mCAR). The latter is highly expressed in the developing brain and could thus determine high NSCs susceptibility to CoxB infection at this stage [85,144].

Moreover, CoxB can lead to NSC apoptosis through the induction of the innate immune response and/or infiltrating immune cells. Interestingly, CoxB does not seem to infect astrocytes and infection of partially differentiated NSCs induces the release of abundant extracellular microvesicles containing the virus [83].

Due to the high tropism for NSCs, possible persistence within the cells and induction of apoptosis [82,84], CoxB may impair normal CNS development and alter neuronal migration (Figure 2) [85]. The majority of studies were performed with cellular systems derived from neonatal/fetal mouse or human cell lines like neuroblastoma, astrocytoma, and glioblastoma. A reliable model of NSCs would be relevant for a better understanding of viral molecular pathways and testing new drugs [150].

## 3. TORCH Pathogens Effects on NSC Neuronal and Glial Derivatives

### 3.1. ZIKV

**Figure 3:**
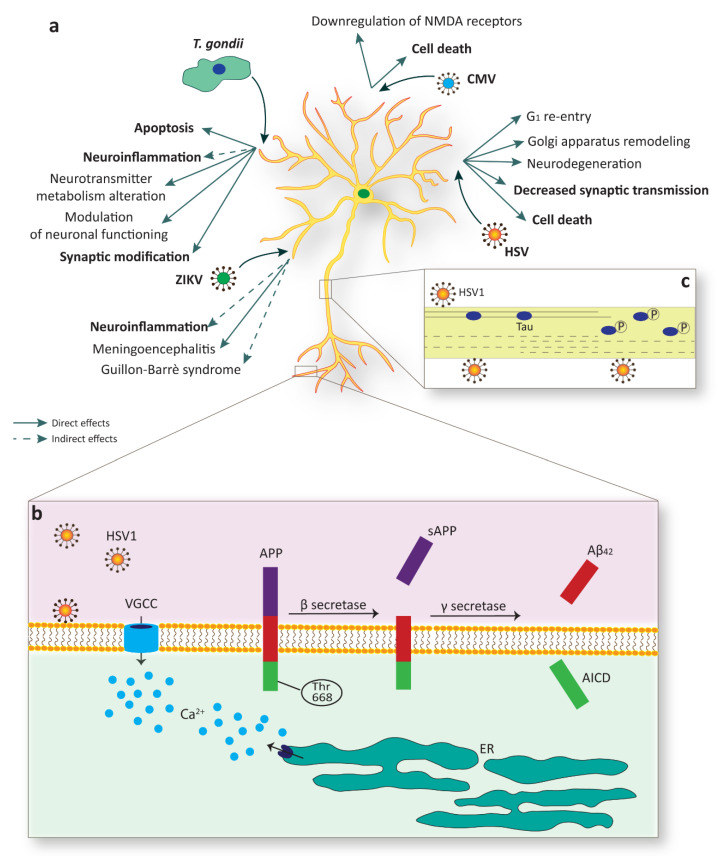
Mature neurons are a target of specific TORCH agents. (**a**) Multiple infections sustained by different pathogens, such as Zika virus (ZIKV), *Toxoplasma gondii* (*T. gondii*), cytomegalovirus (CMV), and Herpes simplex virus (HSV), evoke detrimental effects in mature neurons, including cell death or substantial alterations in function. (**b**) HSV-1 infection sustains membrane depolarization with the consequent increase in intracellular calcium via voltage-gated calcium channels (VGCC) or through depletion from intracellular stores, such as the endoplasmic reticulum (ER). HSV-1 infection is also linked to amyloid precursor protein (APP) phosphorylation of Thr668 which facilitates the amyloidogenic APP cleavage by BACE1 (as known as β secretase) and the release of Aβ_42_. AICD, APP intracellular domain. (**c**) A paradigmatic example of structural alteration due to viral infection is linked to the phosphorylation of the microtubule-associated protein MAPT (as known as Tau). Tau phosphorylation upon HSV-1 infection produces its release from microtubules with consequential loss in stability.

Although ZIKV has been shown to infect the adult brain, in the most cases the infection is asymptomatic or with mild symptoms. Nevertheless, in rare cases adult ZIKV infection can result in meningoencephalitis and Guillain–Barré syndrome (GBS), an immuno-mediated disease occurring post-infection [151,152,153,154,155,156]. The GBS is likely responsible of the acute flaccid paralysis (AFP) induced by ZIKV. Here, the individual manifests progressive weakness (lasting hours or days). In general, *Flaviviruses* can cause AFP in two ways: the first is direct infection of spinal cord with damage of motor neurons and the second is GBS. This distinction is clinically important because, even though there is almost a total overlap of symptoms, the therapy is different, i.e., immunosuppressants eligible for GBS treatment are contraindicated in myelitis (Figure 3a) [41].

Another rare serious complication of ZIKV infection in adult individuals is meningoencephalitis, a medical condition that resembles both meningitis and encephalitis, which is an infection or inflammation of the meninges and brain [42,43]. This shows that neurotropism of ZIKV is not limited to proliferating NSCs but can target adult and differentiated neurons too, even if with a lower efficiency (Table 1) [23,39].

### 3.2. Toxoplasma

This zoonotic parasite can persist lifelong in the CNS within neurons, modifying their function and structure and thus leading to specific behavioral changes of the host [157,158,159]. The parasite can induce direct modifications in the infected cells through different mechanisms, for example by altering dopamine metabolism, by functionally silencing neurons, as well as by hindering apoptosis [46]. Moreover, indirect effects of the peripheral immune system and alterations of the immune status of the CNS, observed during chronic infection, might also contribute to changes in neuronal connectivity and synaptic plasticity [46,160,161,162].

After reaching the CNS, parasites infect a large number of glial cells during the acute phase, in contrast to the low numbers of infected neurons. Subsequently, stage conversion takes place predominantly in neurons resulting in bradyzoite-filled cyst development. Although neurons also produce some cytokines, they lack specific intracellular mechanisms to inhibit parasite growth, which provides an explanation for the relatively high number of infected neurons during the chronic stage [163,164,165,166,167,168].

Serious toxoplasmic encephalitis can develop upon reactivation of the latent infection in immunocompromised hosts. In the latent phase, the slowly replicating bradyzoites can persist lifelong inside neurons. Hence, the infected neurons interfere directly or indirectly with non-infected near and distant neurons, potentially leading to altered neuronal function and to behavioral and psychiatric diseases (for more comprehensive information, see review Parlog et al., 2015 [46]).

For what concerns the direct influence of *T. gondii* on host cells, it has been studied in many in vitro and in vivo systems and various mechanisms have been proposed: (i) the capacity of the parasite cysts to disturb dopamine metabolism. Indeed, Prandovszky and colleagues found that *T. gondii*’s genome encodes for tyrosine hydroxylase and there is a three-fold increase in the amount of dopamine released in infected cells compared to uninfected cells [47]. These results lead to the hypothesis that an excess of dopamine produced by the parasite could interfere with crucial brain functions. However, the information provided by the limited number of studies on this topic, in animal models, is not conclusive and even contradictory [169]; (ii) the direct modulation of neuronal functions thanks to active influence on calcium signaling upon glutamate stimulation, thus leading to either hyper- or hyporesponsive neurons; (iii) Alterations in apoptosis have been considered, even though there are still conflicting opinions. Some studies suggest that *T. gondii* blocks apoptosis by inducing anti-apoptotic genes upregulation in the host cell or by modulating the apoptotic signaling pathway (Figure 3a) [170,171,172,173]. On the other hand, other reports indicate that *T. gondii* promotes apoptosis on mouse NSCs, as discussed before.

The influence of *T. gondii* on CNS occurs even with indirect effects. Indeed, in chronic infection neuroinflammation occurs with permanent activation of resident glia in all CNS and the recruitment of peripheral immune cells. Most of the inflammatory mediators are potentially toxic for neurons and can cause neurodegeneration, neurotransmitter alterations, or trigger abnormalities in the morphology and functionality of neurons [48,49,50]. Abnormalities related to neurotransmitter release involve cytokines and inflammatory mediators such as IFN-c, TNF, NO, IL-1, and IL-6, all commonly present during toxoplasmic encephalitis. These may affect neurotransmitters via (i) activation of indoleamine-2,3-dioxygenase (IDO) enzyme, (ii) activation of MAPK, (iii) alteration in tetrahydropterin (BH4) enzyme activity, and (iv) excitotoxicity and oxidative stress [160]. In particular, the increase of IDO expression leads to the degradation of tryptophan, the amino acidic precursor of serotonin [174,175]. Moreover, MAPK is involved in a plethora of pathways, including the activation of serotonin transport and the inhibition of dopamine recycle. On the other hand, BH4 enzyme is involved in the synthesis of neurotransmitters like serotonin, dopamine, and norepinephrine [176]. Chronic *T. gondii* infection also triggers changes in protein expression of pre- and post-synaptic compartments of the mature neurons, thus impairing neuronal structure, reducing dendritic complexity, and inducing modifications in dendritic spine number, distribution, and morphology (Figure 3a) [46,51].

Some studies conducted in mice with chronic *T. gondii* infection show behavioral alterations [46,52]. This could be due to the high tropism of parasite cysts in the amygdala [177] and induction of neurotransmitters dysregulation [46]. In humans, latent infection with *T. gondii* has generally been considered of little clinical relevance. In 2001, the first report on the potential impact of *T. gondii* on psychomotor performance suggested that individuals with latent infection exhibited increased reaction times and they lose their concentration more quickly [178]. However, a recent study by Stock and colleagues revealed a paradoxical improvement of cognitive control process in *T. gondii*-infected healthy individuals that is a positive effect of infection on cognitive capacities of the host [179]. Moreover, another study performed in a murine model of Alzheimer’s disease (AD) suggests that infection with *T. gondii* inhibits neuronal degeneration (Table 1) [180].

Interestingly, classical psychiatric diseases such as schizophrenia, mood disorders, psychosis, and self-directed violence are also linked to toxoplasmosis, since *Toxoplasma* seropositivity was elevated in psychiatric patients compared to heathy volunteers in numerous studies [53,54,181,182,183,184,185].

One of the most ‘convenient’ explanations for the behavioral and neuropsychological deficits is that the parasite directly infects neurons interfering with their survival and function. In particular, a possible mechanistic explanation for the association between *T. gondii* infection and psychiatric illness concerns the cytokine-mediated activation of IDO enzyme. Tryptophan degradation by IDO leads to increased concentrations of two neuroactive metabolites, quinolinic acid (QA), and kynurenic acid (KA). In human patients, excessive QA and KA levels have been correlated with a number of neurodegenerative disorders, depression, and schizophrenia. Produced primarily by microglia, QA binds to glutamate N-methyl-D-aspartate receptors (NMDARs) inducing excitotoxicity and oxidative stress in the brain [46,186,187]. Instead, KA, produced primarily in astrocytes, is a potent antagonist of NMDARs, leading to disturbances in glutamatergic and dopaminergic neurotransmission.

### 3.3. CMV

CMV can infect differentiated neural cells, too [61,188]. However, differential susceptibility of neuronal subtypes to CMV infection and the mechanisms through which this occurs is still a matter of debate [59,60,62,116,189]. One issue is related to the kinetics of infection. It is possible that infection in neurons proceeds more slowly, they survive more than astrocytes and appear to be quite refractory to morphological changes that normally occur during permissive infection [116]. Another issue is related to the increased susceptibility of some specific neuronal cell types. Few neuronal subtypes expressing viral antigens may support CMV replication; in fact stable expression of host viral IE genes is obtained in cholinergic neurons, while glutamatergic, GABAergic, and noradrenergic neurons show relatively low gene expression and are therefore less vulnerable [65,190,191]. In few infected neuronal cells, CMV induces apoptosis and appears to impair neuronal function, as suggested by the downregulation of the NMDA receptor (Figure 3a) [65].

### 3.4. HSV

HSV-1 and 2 establish lifelong persistent infection in the host, as reported in the previous section. However, new studies also show a close relationship among HSV and neurodegeneration, in particular with AD. Several reports have described HSV infection in mature adult neurons and, to this purpose, a wide variety of models has been applied. Some studies have been performed on cortical or trigeminal neurons derived from human iPSCs [73,192] or from adult mice [72,193]. Human iPSC-derived cerebral organoids have also been used to better model the interaction of HSV with the complex cellular and architectural structure of the human CNS [194,195]. All these in vitro models have contributed to elucidate the mechanisms and the consequences of HSV infection according to various neuronal types and subtypes, and to their interactions with environment [196]. For example, Zimmer et al. (2018) [192] exploited human iPSC-derived cortical and trigeminal neurons to show a higher sensitivity of trigeminal neurons compared to cortical cells and the importance of TLR3 (Toll-Like Receptor 3) in this phenomenon. They hypothesized that the selective permissiveness might elucidate why trigeminal neurons may act as reservoir of the virus during latent infections [140].

Despite such a broad range of models, HSV toxicity on neurons is not yet fully understood. In particular, aspects related to the choice between viral latency and lytic replication and the correlation with cell cycle are still uncertain. Indeed, terminally differentiated neurons are in G_0_ phase, but stress stimuli have been shown to induce re-entry into G_1_ [197,198]. It has also been suggested that G_0_-arrested neurons stimulated to re-enter the cell cycle undergo apoptosis [68,69,70] and that activation of G_1_ cyclin/CDK activity results in neuronal cell death via apoptotic pathways [71,199]. Moreover, transition of neuronal cells from G_0_ to G_1_ can induce expression from IE promoters on latent viral genomes [200,201], which may be one of many possible mechanisms leading to re-initiation of the lytic cascade. To this regard, a study by Hobbs and colleagues shows that IE genes induce the cell cycle arrest, thus hypothesizing that this event may serve a dual function, i.e., (i) to activate viral gene expression by promoting a permissive intracellular milieu and (ii) to prolong infected-neuron metabolism [133].

Another study by Martin and colleagues, using mouse cortical neurons, observed that alteration of Golgi apparatus in neurons is an early and probably irreversible event that could potentiate the neurodegenerative process [72]. This hypothesis stems from the fact that Golgi and endoplasmic reticulum are involved in production of neurotransmitters and proteins that are released during synaptic activity in neurons and the virus could modulate diverse cell signaling pathways leading to Golgi apparatus remodeling. However, the exact molecular mechanisms and the functional consequences to neurons are currently unknown [202].

Furthermore, D’Aiuto and colleagues reported changes in architecture and functional activity on human iPSC-derived neurons after HSV infection and functional brain fluctuations leading to working memory impairments [73]. Accordingly, Piacentini and co-workers (2015) [74] observed a reduction of Synapsin-1 and Synaptophysin expression and a decrease of synaptic transmission on mice cortical neurons after HSV infection (Figure 3a).

### 3.5. HSV-1 Infection and Alzheimer’s Disease Neurodegeneration

AD is an incurable, unremitting, neurodegenerative disorder characterized by a progressive cognitive decline affecting short-term memory and, at later stages, language, mood, movement, and physiological functions. AD is associated with the accumulation of insoluble forms of amyloid-β (Aβ) plaques in extracellular spaces and aggregation of microtubule protein MAPT (as known as Tau) in neurofibrillary tangles within neurons [203]. Aβ originates from the proteolytic cleavage of amyloid precursor protein (APP) by a complex family of enzymes (γ- and β-secretases), which includes presenilin 1 (PSEN1) and PSEN2. Mutations in the genes encoding for *APP*, *PSEN1*, and *PSEN2* are responsible for an autosomal dominant form of the disease (familial AD, fAD), which typically strikes less than 1% of the patients [204]. It is widely believed that these autosomal dominant mutations lead to an amyloidogenic shift in the cleavage of APP resulting in the favored generation of the Aβ_42_ isoform over the smaller, less hydrophobic, Aβ_40_ [205]. Aβ_42_ has a strong propensity to precipitate in vivo, and forms extracellular inclusions, which eventually transit into senile plaques (Figure 3b).

Sporadic AD (sAD), on the other hand, is the most common form of the disease and typically develops after 60–65 years of age. Even though the environmental events triggering sAD are still unknown, several risk factors have been identified [206,207,208,209,210]. Among these, the polymorphism associated with the gene encoding for apolipoprotein E (*APOE*) is the most-well characterized and has been unequivocally established as the most important susceptibility gene for sAD [211]. Compared to the *APOE3* allele, which appears to be neutral in terms of risk of developing dementia, *APOE2* has been reported to be protective [212], while *APOE4* increases LOAD risk by approximately 3-fold for heterozygous carriers, and by 15-fold for homozygous carriers [213].

Various agents and viruses in particular, have been proposed as potential causes of AD. In fact, several studies have demonstrated that hippocampal cells are highly susceptible to infection by several viruses, including Cox virus, borna disease virus, HIV, CMV, and ZIKV [214,215,216,217,218]. Among potential infectious candidates, HSV-1 has attracted much attention because it is neurotropic, ubiquitous in the general population, and able to establish life-long latency in the host. It is known that HSV-1 establishes a latent infection in the trigeminal ganglion of infected subjects and, following periodic reactivations triggered by stress factors (i.e., hyperthermia), newly produced viral particles are transported back to the original site of infection, causing blisters on orolabial mucosa (see above). Viral particles may also reach the CNS by travelling along the fibers of the trigeminal ganglion neurons that project to trigeminal nuclei located in the brainstem. From here, the virus has been shown to travel to the thalamus and finally to the sensory cortex where it causes acute events like encephalitis (herpes simplex encephalitis, HSE), or chronic latent infection within the CNS [219,220]. First relevant observations advocating for a possible link between HSV-1 and AD were made in the early 80s when it was found that individuals surviving HSE showed clinical signs reminiscent of AD, and that brain regions primarily affected by HSE (hippocampus, frontal and temporal cortices) were the same regions compromised in AD [221]. Subsequent findings corroborated these initial observations by providing evidence of HSV-1 DNA presence in the brain of a high fraction of elderly people (about 60%) and, more importantly, in amyloid plaques of AD patients [78,222,223]. On these premises, the possibility that HSV-1 infection could play a role in the development of sAD was an intriguing hypothesis and it definitely encouraged a large number of studies. The confirmation of an actual link between sAD and viral infections came from two independent groups who reported an increased susceptibility of *APOE4* carriers to contract HSV-1 and a higher risk of developing AD for patients with an *APOE4* genotype and a full-blown HSV-1 infection [223,224].

A number of possible mechanisms linking HSV-1 and increased AD risk has been proposed. Several in vitro studies demonstrated that HSV-1 interferes with APP processing leading to an accumulation of diverse intra- and extracellular APP fragments, including Aβ_40_ and Aβ_42_ [75,76,77]. Additionally, HSV-1 has also been reported to bind neuronal membrane and sustain prolonged neuronal excitability and firing of action potentials with a consequent significant increase in intracellular calcium [77]. Dysregulation of calcium homeostasis not only elicits neurodegeneration per se, but it is also responsible for the phosphorylation of APP on Thr668, a critical event in the amyloidogenic cleavage of APP and Aβ formation (Figure 3b) [225].

HSV-1 has also been shown to contribute to Tau pathology in AD. Protein Tau is known for its stabilizing effects on neuronal cytoskeleton and its phosphorylation produces the detachment from cytoskeletal microtubules, with a consequent loss in stability, reduction in dendritic spine density, and neuronal arborization [226]. Hyperphosphorylated Tau is the main component of neurofibrillary tangles and HSV-1 contributes to its phosphorylation on multiple sites by acting on GSK3β and PKA [78]. Another event that may promote the kinetics of Tau aggregation is its caspase-mediated cleavage. Similarly to what was observed for Tau phosphorylation, cleaved Tau has been detected in human cells a few hours after HSV-1 exposure, thus supporting the idea that the virus might facilitate neurodegeneration through diverse simultaneous mechanisms (Figure 3c) [79].

Detrimental effects sustained by Aβo do not only alter synaptic functions in mature neurons, but evidence suggests that NSCs and adult neurogenesis processes may also be affected [227,228]. Adult neurogenesis in the hippocampus is critically involved in the memory and learning processes [229], therefore the identification of agents that may interfere with this process may be relevant in the study of the disease. To this regard, recent findings by Li Puma and colleagues have shown that HSV-1 reduces hippocampal NSC proliferation in vitro and in vivo and decreases neuronal differentiation in favor of the acquisition of glial identity [67]. Similarly to what was described by Piacentini and colleagues, APP fragmentation and Aβ_42_ formation were also observed in adult hippocampal NSCs with consequent oligomer aggregation, both in vitro and in vivo [67].

Very recently, Palamara’s group proposed a mouse model of recurrent HSV-1 infections which recapitulates reiterated viral activations, closely recapitulating what happens in humans [80]. Results provided clear evidence that recurrent viral activations, obtained by exposing infected mice to hyperthermia conditions, can cause viral replication in the brain leading to Aβ_40_ and Aβ_42_ accumulation in the cortex and in the hippocampus, Tau phosphorylation and cleavage, and increase of proinflammatory cytokines [80]. Of note, authors also reported that accumulation of AD hallmarks was paralleled by cognitive decline, which became progressively more significant with a higher number of viral reactivations [80]. These results provide novel insights in the pathophysiology of AD, demonstrating that multiple HSV-1 activations in the host, a common condition in humans, represent a risk factor for AD. With the most recent advance in 3D cell models, findings supporting a link between HSV-1 infection and AD have also been validated in human cerebral organoids. An elegant work by Cairns and colleagues has recently elucidated HSV-1 infection in human brain organoids, demonstrating the role of HSV-1 in sustaining Aβ plaque accumulation, gliosis, neuroinflammation, and alteration of neuronal electrical properties in absence of any exogenous mediator of AD [81]. The authors also provide proof of VCV, a commonly used antiviral agent for HSV-1 infection, as an effective drug in limiting HSV-1 induced neurotoxicity and gliosis, thus underlying the role of the virus in sustaining AD major hallmarks (Table 1).

Based on all the elements supporting an actual link between HSV-1 and AD, clinical trials have started to explore potential beneficial effects of anti-viral drugs in the treatment or prevention of AD-related dementia. Retrospective studies already proved that antiviral treatment reduces the risk of AD in a large population study in Taiwan, suggesting that active treatment of HSV-1 infection at an early age can prevent the occurrence of AD in the elderly [230]. Almost simultaneously to the collection of this data, in 2018, a phase II study supported by NIH (NCT03282916) started recruiting adults with mild AD who also tested positive for HSV-1 or HSV-2 to evaluate the efficacy of antiviral drug valacyclovir in slowing or preventing symptoms. Results collected from these ongoing clinical investigations will be determinant for evaluating potential applications of anti-viral therapies for the treatment of AD.

## 4. Conclusions

TORCH-infections represent a major challenge, but also an opportunity for the scientific community to collaborate across disciplines and learn fundamental principles of virology, stem cell biology, and human neurodevelopment and neurodegeneration. Stem cell-based modeling of TORCH exposure, in particular to ZIKV, is one of the clearest examples of how the promise of human stem cell research may be realized and how decades of advances in basic stem cell biology allowed researchers to act immediately in response to a global health emergency.

Human neural stem cells appear to be very vulnerable to pathogens, by altering the orchestrated mechanisms of proper neurodevelopment. Evidence from varying sources investigating multiple model systems all consistently point to the conclusion that vertical transmission of TORCH pathogens, from pregnant women to their unborn fetuses via the trans-placental route, targets NSCs in the developing brain. This can cause death, apoptosis, and decreased proliferation of these cells, and results in impaired brain development and therefore a microcephalic phenotype. The recent outbreak of ZIKV highlights the need not only to better understand how ZIKV and other TORCH pathogens breach the placental barrier and induce congenital disease, but also the urgent need to assess the safety and efficacy of antimicrobial therapeutics in pregnant women.

On the other side, we learnt that insults to neural cells may produce unexpected long-term consequences in adult neurons, impacting their health and survival and triggering neurodegenerative disorders. HSV-1 infection and correlation to AD represents a paradigmatic example that may open the door to other examples.

Future modeling of the TORCH-pathogen infection will address the extent of the associated pathophysiology and the underlying biological mechanisms, as well as furthering the development of platforms to facilitate drug screening.

## Figures and Tables

**Table 1:** Summary of the main studies addressing TORCH pathogens’ effects on neural progenitor cells (NPCs), neural stem cells (NSCs), and their progeny. ER: endoplasmic reticulum.

Pathogen	Targets	Effects	Models	References
ZIKV	NPCs	AKT-mTORC pathway inhibition	Human fetal NSCs	[36]
		STING cleavage	Human fibroblasts	[37]
		SEPTIN2 cleavage	NPCs obtained from human H9 ESC-derived embryoid bodies	[38]
		Centrosomal aberration	Human neocortical NES cells	[23]
		pTBK1 relocation	Human neocortical NES cells	[23]
		Proliferation impairment	Human iPSC-derived forebrain organoids	[39,40]
		Radial scaffold disorganization and architectural impairment	Human organotypic fetal brain slices, post-mortem fetal brain samples	[23]
		Cell death	Human iPSC-derived forebrain organoids	[39,40]
	Neuronal progeny/Immune cells	Acute flaccid paralysis: (i) damage of motor neurons, (ii) Guillan–Barrè syndrome	/	[41]
		Meningoencephalitis	/	[42,43]
*T. gondii*	NPCs	Gliogenesis induction	Mouse NPCs	[44]
		Neurogenesis impairment	Mouse NPCs	[44]
		ER stress dependent apoptosis	Mouse NSCs	[45]
	Neuronal progeny	Apoptosis	/	[46]
		Neurotransmitter metabolism alteration	Neural cells in mouse brain tissue	[46,47]
		Neuroinflammation	/	[48,49,50]
		Synaptic modification	Mouse model	[46,51]
		Behavioral alterations and psychiatric diseases	Primary human temporal-lobe NSC lines	[46,52,53,54]
Rubella Virus	NPCs	Cell death	Autoptic fetal tissue	[55]
	Neuronal progeny	Cell death	Autoptic fetal tissue	[55,56,57]
CMV	NPCs	Neurogenesis impairment	Human iPSC-derived brain organoids	[58]
		Migration impairment	Human iPSC-derived brain organoids	[58]
		pTBK1 relocation	Human neocortical NES cells	[23]
		Proliferation impairment	Human fetal brain-derived NPCs; human fetal NES cells; hNPCs	[59,60,61]
		Cell death	Primary human neuronal cell cultures; human fetal NES cells; hNPCs; human iPSC-derived brain organoids	[58,59,61,62,63]
		Dysregulation of genes involved in multipotency, modulation of cellular excitability and calcium signaling	Human iPSC-derived NSCs; hNPCs; human iPSC-derived brain organoids	[58,64,65]
		SOX4, DCX, Nestin, SOX2 and GFAP proteasomal degradation	Human fetal brain-derived NPCs; hNPCs	[60,64]
	Neuronal progeny	Apoptosis	Human iPSC-derived NSCs	[65]
		Downregulation of NMDA receptor	Human iPSC-derived NSCs	[65]
HSV	NPCs	Proliferation surge (in acute phase)	Mouse NSCs	[66]
		Proliferation impairment (in chronic phase)	Mouse NSCs	[66]
		Neurogenesis impairment (in chronic phase)	Mouse NSCs	[66]
		APP fragmentation	Mouse adult hippocampal NSCs	[67]
		Gliogenesis induction	Mouse adult hippocampal NSCs	[67]
	Neuronal progeny	G_1_ re-entry stimulated apoptosis	Cerebellar granule cells; rat dorsal root ganglion neurons; human neuronal cell line; rat sympathetic neurons	[68,69,70,71]
		Golgi apparatus remodeling	Mouse cortical neurons	[72]
		Changes in architecture and functional activity	Human iPSC-derived neurons	[73]
		Decrease of synaptic transmission	Mouse cortical neurons	[74]
		Accumulation of APP fragments	Primary cultures of cortical neurons from rat embryos; mouse brains	[75,76,77]
		Increase of intracellular calcium	Rat cortical neurons	[77]
		Increase of Tau phosphorylation and cleavage	Mouse fetal neurons; AD brain specimens	[78,79]
		Neuroinflammation	Human brain organoids; mouse models	[80,81]
CoxB	NPCs	Cell death	Neonatal mice brain; mouse cortical NPCs	[82,83,84]
		Migration and neurogenesis impairment	Neonatal mice central nervous system	[85]
